# Vitamin D ameliorates prediabetic cardiac injure via modulation of the ErbB4/ferroptosis signaling axis

**DOI:** 10.3389/fimmu.2025.1626295

**Published:** 2025-07-17

**Authors:** Yufan Miao, Yujing Zhang, Luoya Zhang, Hao Chen, Lulu Tang, Wenjie Li, Chenxi Gu, Lili Lang, Xing Li, Hanlu Song

**Affiliations:** ^1^ Department of Nutrition and Food Hygiene, College of Public Health, Zhengzhou University, Zhengzhou, Henan, China; ^2^ Department of Orthopedics, The First Affiliated Hospital of Zhengzhou University, Zhengzhou, Henan, China; ^3^ Biomedical Engineering Department, Changzhi Medical College, Changzhi, Shanxi, China; ^4^ Department of Hematology, The First Affiliated Hospital of Zhengzhou University, Zhengzhou, Henan, China; ^5^ School of Design and Innovation, Tan Kah Kee College, Xiamen University, Zhangzhou, China

**Keywords:** prediabetes, vitamin D, ErbB4, ferroptosis, myocardial injury

## Abstract

Vitamin D (VD) deficiency is closely associated with metabolic health and cardiac function in prediabetic patients, yet its underlying mechanisms remain unclear. This study investigated the role of VD intervention in prediabetic cardiac injury through *in vivo* and *in vitro* models, with particular focus on the ErbB4/ferroptosis axis. Using a high-fat diet-induced KKAy prediabetic mouse model, we observed significant metabolic abnormalities (increased body weight, hyperglycemia, insulin resistance) and cardiac remodeling (cardiac hypertrophy and functional impairment) (*P*<0.05). Remarkably, 16-week vitamin D (VD_3_) supplementation substantially ameliorated these pathological changes and reduced serum cardiac injury markers (*P*<0.05). Mechanistic studies revealed that VD_3_ downregulated myocardial NRG1 expression, inhibited ErbB4 phosphorylation (p-ErbB4) and YAP activation (p-YAP), while reversing the abnormal expression of ferroptosis-related proteins. *In vitro* experiments confirmed that high glucose combined with palmitic acid (HGPA) induced ferroptosis in H9c2 cardiomyocytes, which was alleviated by 1,25(OH)_2_D_3_ intervention through suppression of ErbB4 phosphorylation. Notably, combined treatment with 1,25(OH)_2_D_3_ and the ErbB4 phosphorylation inhibitor dacomitinib demonstrated synergistic protective effects. Our findings not only expand the understanding of the association between prediabetes and VD, but also reveal a relationship between ErbB4 and cardiac ferroptosis in prediabetic conditions.

## 1 Introduction

Cardiovascular complications represent a major cause of morbidity and mortality in diabetic patients, with cardiac dysfunction emerging as a particularly devastating consequence of chronic metabolic dysregulation (1). The pathophysiological triad of hyperglycemia, insulin resistance, and compensatory hyperinsulinemia creates a vicious cycle that progressively impairs cardiac function, ultimately culminating in structural abnormalities including myocardial hypertrophy, fibrosis, and both diastolic and systolic dysfunction (2, 3). Despite the clinical significance of diabetic cardiomyopathy, current therapeutic options remain limited, highlighting the urgent need for novel intervention strategies.

Emerging evidence suggests that the path to diabetic heart failure may begin during the prediabetic stage, characterized by early manifestations of left ventricular hypertrophy and diastolic dysfunction (4, 5). This preclinical phase represents a critical therapeutic window, as metabolic and cardiac abnormalities at this stage may still be reversible through timely intervention, in contrast to the largely irreversible damage observed in established diabetes. Among potential interventions, vitamin D (VD) has attracted considerable attention due to its pleiotropic effects on glucose metabolism and cardiovascular function (6–8). Epidemiologic studies have demonstrated that VD supplementation can reduce the risk of diabetes progression in prediabetic individuals by up to 46%, while mechanistic research has revealed multiple cardioprotective pathways mediated through the vitamin D receptor (VDR) (9–11). However, the specific benefits of VD intervention during the prediabetic phase, particularly regarding early cardiac protection, remain poorly understood.

At the cellular level, the pathogenesis of diabetic cardiomyopathy involves complex interactions between metabolic stress and oxidative damage (12). The diabetic milieu, characterized by chronic hyperglycemia and hyperlipidemia, promotes excessive generation of reactive oxygen species (ROS) and lipid peroxides, creating conditions conducive to ferroptosis - an iron-dependent form of regulated cell death that may play a pivotal role in myocardial injury (12–14). Interestingly, recent studies have implicated the epidermal growth factor receptor ErbB4 as a potential mediator between metabolic stress and cardiomyocyte survival (15, 16). While the NRG1/ErbB4 signaling axis is known to regulate cardiac remodeling, and high glucose conditions have been shown to upregulate NRG1 expression, the potential crosstalk between ErbB4 signaling and ferroptosis in the diabetic heart remains unexplored (15, 17–19).

To address these critical questions, we employed a comprehensive experimental approach combining *in vivo* and *in vitro* models of prediabetic cardiac injury. Using high fat diet (HFD) -fed KKAy mice and high glucose/palmitic acid (HGPA)-treated H9c2 cardiomyocytes, we systematically investigated (1): the cardioprotective effects of VD intervention during the prediabetic stage; (2) the involvement of ErbB4 signaling in metabolic stress-induced cardiac injury; and (3) the potential interplay between ErbB4 activation and ferroptosis regulation in the diabetic heart. Our findings provide novel insights into the molecular mechanisms underlying early diabetic cardiomyopathy and identify potential targets for preventive intervention.

## 2 Materials and methods

### 2.1 Chemical reagents and antibodies

VD_3_ was procured from Sigma Aldrich (St. Louis, MO, USA). Dulbecco’s modified eagle medium (DMEM) and fetal bovine serum (FBS) were obtained from GIBCO (Grand Island, NY, USA) and LONSERA (Auckland, New Zealand), respectively. 1,25(OH)_2_D_3_ and Ferrostatin-1(Fer-1) were purchased from GLPBIO (Montclair, CA, USA), and Dacomitinib (Dac) was acquired from MedChemExpress (Monmouth Junction, NJ, USA). The sources of antibodies were as follows: NRG1 (10527-1-AP), SLC7A11 (26864-1-AP), TFR1 (84766-4-RR), NCOA4 (83394-4-RR), β-actin (20536-1-AP) were purchased from Proteintech (Wuhan, China), ErbB4 (RT1276), YAP (RT1664), Phospho-YAP (ET1611-69), ACSL4 (ET7111-43), Ferritin (R1601-9), GPX4 (ET1706-45) were purchased from HUABIO (Hangzhou, China). Phospho-ErbB4 (Tyr1284) (AF3445) were purchased from Affinity Biosciences (Cincinnati, USA).

### 2.2 Animals and Grouping

Four-week-old specific pathogen free (SPF) male C57BL/6J mice (n=12) and KKAy mice (n=48) were purchased from Beijing Huafukang Bio-technology Co. The animals were housed in a controlled barrier facility with a 12h light/dark cycle at 22 ± 1°C and 55 ± 5% humidity, with ad libitum access to food and water. Following a 2-week acclimatization period, C57BL/6J mice were designated as the normal control group (NC) and fed a standard chow diet (fat: 4.0 gm%), while KKAy mice received a HFD (17.9 gm%) for 6 weeks to establish a prediabetic model. The modeling criteria were defined as fasting blood glucose (FBG) levels maintained within 6.7 to 16.7 mmol/L, combined with 2h post-oral glucose tolerance test (2h-OGTT) blood glucose levels ranging from 11.1 to 30 mmol/L. Successfully modeled KKAy mice were randomly divided into four groups according to body weight and FBG levels: model group (MC), low-dose VD_3_ intervention group (LVD, 0.42 IU/g/w), medium-dose VD_3_ intervention group (MVD, 1.68 IU/g/w), and high-dose VD_3_ intervention group (HVD, 4.20 IU/g/w). VD_3_ dissolved in 0.1 mL corn oil was administered via intraperitoneal injection, and the NC and MC groups received an equivalent volume of corn oil. After 16 weeks of VD_3_ intervention, mice were anesthetized by intraperitoneal injection of sodium pentobarbital at a dose of 50 mg/kg. Heart tissues and blood samples were then immediately collected and preserved for further analysis. All animal experiments were approved by the Life Sciences Ethics Review Committee of Zhengzhou University (Ethics No. ZZUIRB2021-GZR0141).

### 2.3 Echocardiographic measurement

After 16 weeks of VD_3_ intervention, echocardiography was performed by using Vevo2100 imaging system. Parasternal short-axis M-mode echocardiographic images were obtained at papillary muscles level. The following parameters were recorded: left ventricular (LV) mass, left ventricular volume at diastole and systole (LV Vol;d and LV Vol;s), left ventricular internal dimension at diastole and systole (LVID;d and LVID;s), left ventricular ejection fraction (EF), fractional shortening (FS), and interventricular septal thickness at systole and diastole (IVS;s and IVS;d).

### 2.4 Biochemical analysis

FBG, total cholesterol (TC), low-density and high-density lipoprotein cholesterol (LDL-C and HDL-C), creatine kinase (CK) and lactate dehydrogenase (LDH) levels were measured using the Nanjing JianCheng Assay Kits. Serum fasting insulin (INS) and 25(OH)D were measured using Mlbio Enzyme-linked immunosorbent assay Kits.

### 2.5 Measurement of tissue iron, glutathione and malondialdehyde levels

Heart tissue (50 mg) was precisely weighed and placed into a grinding tube containing grinding beads. Subsequently, 450 mL of physiological saline was added, and the tissue was thoroughly ground using a grinder. The resulting mixture was then centrifuged in a pre-cooled centrifuge for 10 min, after which the supernatant was collected for measurement. The protein concentration was determined, and Fe, GSH, and MDA levels were measured as per the provided instructions. Subsequently, values were calculated and analyzed using the designated formulas.

### 2.6 Hematoxylin & eosin, wheat germ agglutinin and Perls prussian blue with DAB staining

Following fixation in 4% paraformaldehyde, cardiac tissues were sequentially subjected to graded ethanol dehydration, xylene clearing, paraffin infiltration, and sectioning into 3-5 μm slices using a rotary microtome. For histological analysis, sections were dewaxed in xylene (3 × 5 min), rehydrated through descending ethanol gradients (100%, 95%, 75%; 2 min each), and stained with H&E per standard protocols. For WGA staining, the sections underwent dewaxing, antigen retrieval, and incubation with primary and secondary antibodies. Simultaneously, the WGA working solution was drop-stained, counterstained with DAPI for nuclear localization, and imaged under a laser scanning confocal microscope. The cross-sectional area of cardiomyocytes was measured using Image J software, and the resulting data were analyzed. Furthermore, sections were deparaffinized, stained with Prussian blue and DAB, and images were observed and captured using a light microscope.

### 2.7 Oil red O and ROS staining

The tissues preserved at -80°C were embedded using OCT compound to prepare frozen sections. Subsequently, these sections were stained with oil red O following the provided guidelines, and the resulting images were observed and captured using a light microscope. For further analysis, the frozen sections were allowed to thaw to room temperature, ensuring moisture control and subsequent drying. Tissue autofluorescence was minimized, and a staining solution for ROS was meticulously added to the sections. After restaining the nuclei with DAPI, the sections were sealed and observed using fluorescence microscopy, with images captured for subsequent analysis.

### 2.8 TUNEL staining

Cardiac paraffin sections were deparaffinized in xylene (3 × 10 min) and rehydrated through a graded ethanol series into distilled water. Antigen retrieval was performed in citrate buffer at 95°C for 20 min. After cooling to room temperature, sections were washed in PBS (3 × 5 min), permeabilized with 0.1% Triton X-100 for 10 min, and treated in 3% hydrogen peroxide in methanol for 20 min in the dark. To suppress lipofuscin autofluorescence, sections were incubated with 10% acetic acid in methanol for 10 min and rinsed in PBS (3 × 5 min). Following equilibration in commercial equilibration buffer, sections were incubated with TUNEL reaction mixture at 37°C for 90 min in a humidified dark chamber. After washing in stop/wash buffer (3 × 10 min), Converter-POD was applied for 30 min at 37°C. DAB chromogen was used for signal development under microscopic monitoring. Sections were counterstained with Mayer’s hematoxylin for 45 sec, dehydrated through graded ethanol, cleared in xylene, and mounted with DPX mounting medium.

### 2.9 Cell cultures and treatment

**Figure 1:**
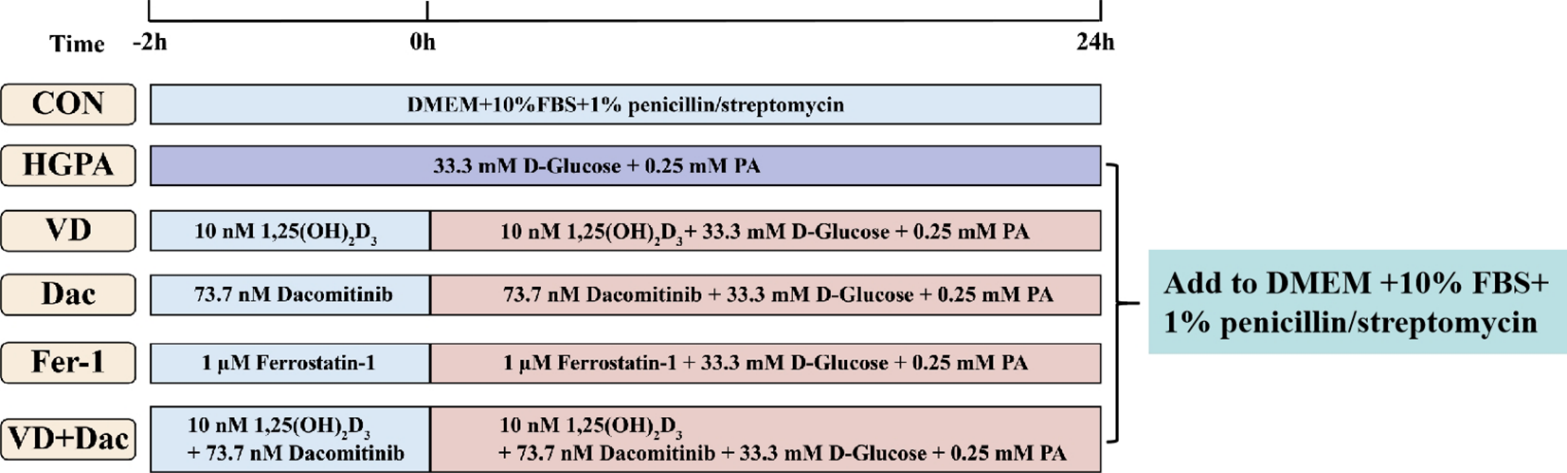
Experimental design of cell experiments.

H9c2 cells were obtained from the First Affiliated Hospital of Zhengzhou University and cultured in DMEM medium supplemented with 10% FBS and 1% penicillin/streptomycin at 37°C in humidified 5% CO_2_ conditions. The cells were used in the experiments once they reached 70-80% confluence. High glucose and palmitic acid medium (HGPA, 33.3 mM d-glucose, 0.25 mM PA) was used to construct injury models by culturing cells for 24h. Addition of 10 nM 1,25(OH)_2_D_3_ and/or 73.7 nM Dacomitinib or 1 μM Ferrostatin-1 pretreated the cells for 2h while co-culturing the cells with HGPA for 24h (**Figure 1**).

### 2.10 Cell viability assay

Cells were seeded in 96-well plates, allowing them to adhere before the addition of medium containing various concentrations of intervening substances. Subsequently, 10 μL of Cell Counting Kit-8 (ApexBio, Houston, USA) solution was introduced into each well, and the plates were incubated at 37°C in an incubator for 1-4h. Absorbance at 450 nm was measured, and cell viability was calculated based on the recorded measurements.

### 2.11 Detection of ROS in cells

Add a minimum of 1 mL of 10 μM DCFH-DA to the cultured cells, enabling its free passage through the cell membrane. Subsequently, intracellular lipase hydrolyzes DCFH-DA into DCFH, which is unable to traverse the cell membrane. Active oxygen within the cell oxidizes DCFH to form DCF, which exhibits fluorescence. The fluorescence intensity of DCF serves as an indicator to measure intracellular active oxygen levels. Incubate the cells for 20 minutes at 37°C in the cell incubator to facilitate the process. Remove any residual DCFH-DA that hasn’t entered the cells, then observe and capture images using a fluorescence microscope.

### 2.12 Western blot analysis

Proteins extracted from murine hearts and cells were initially separated using SDS-PAGE gel and subsequently transferred onto polyvinylidene fluoride membranes. Following this, the membranes were blocked using either 5% skim milk or bovine serum albumin. Later, they underwent incubation with primary and secondary antibodies and were detected using an ECL detection system.

### 2.13 Statistical analysis

The obtained results underwent statistical analysis via SPSS 25.0 software. For quantitative data, normality and tests for normality and variance homogeneity were performed. Quantitative data were presented as mean ± standard deviation (Mean ± SD). Between-group comparisons were performed using ANOVA for one-way multilevel quantitative data and ANOVA for repeated measures across multiple time points. Additionally, the Tukey’s HSD was employed for two-way comparisons between groups, *Р<*0.05 was considered statistically significant.

## 3 Result

### 3.1 VD_3_ supplementation ameliorates VD deficiency and glucolipid metabolism disorders in prediabetic mice

**Figure 2:**
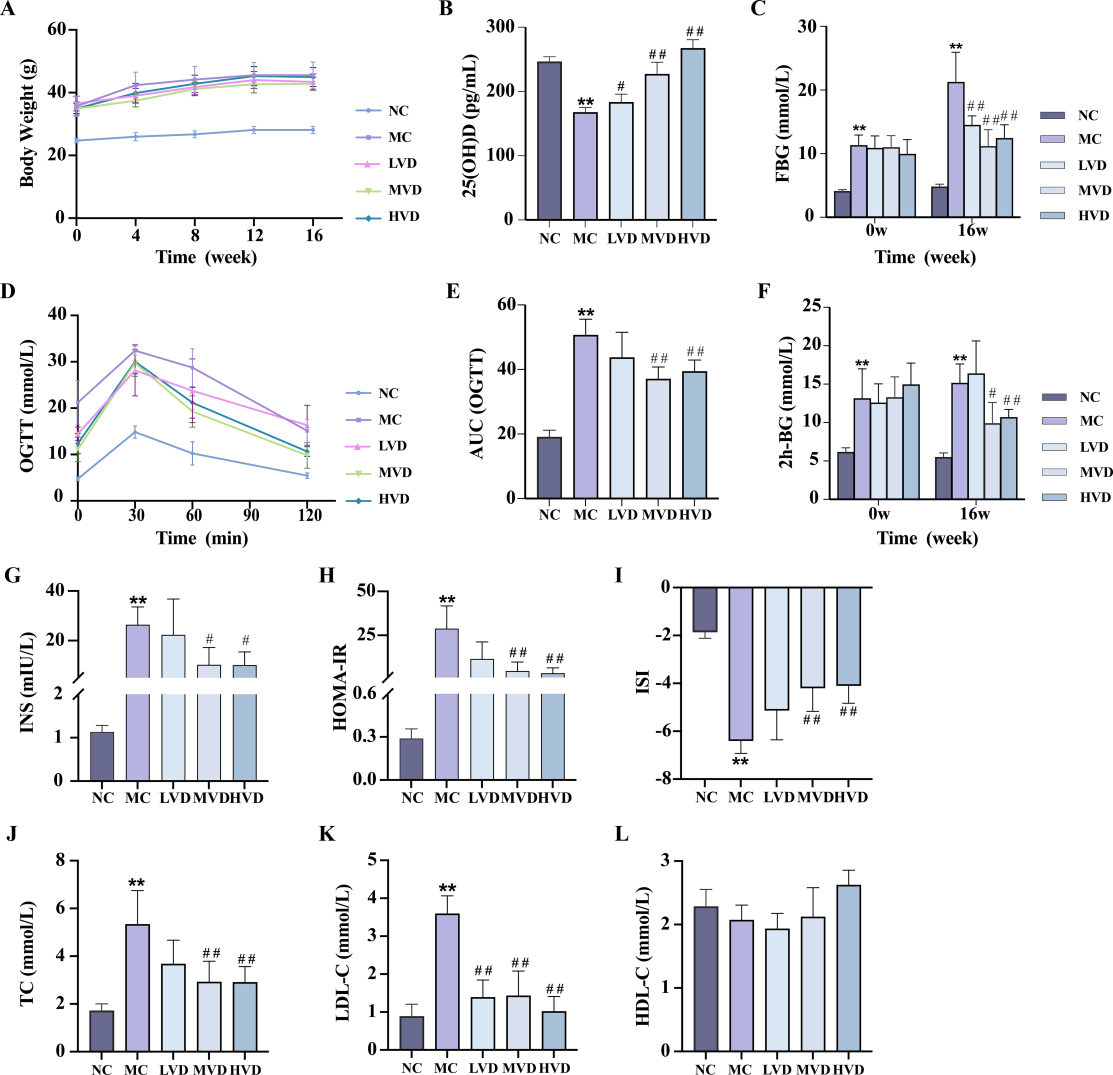
Effects of VD_3_ supplementation on metabolic parameters in prediabetic mice. **(A)** Body weight (n=8). **(B)** Serum 25(OH)D levels (n=5). **(C)** FBG levels (n=8). **(D, E)** OGTT measurements and corresponding AUC (n=8). **(F)** 2h postprandial blood glucose levels (n=8). **(G-I)** Serum fasting insulin and respective insulin indices (n=3-10). **(J-L)** Serum TC, LDL-C, HDL-C levels (n=4). Data are presented as Mean ± SD. ^*^ *P* < 0.05, ^**^ *P* < 0.01, compared with the NC group; ^#^ *P* < 0.05, ^##^ *P* < 0.01, compared with the MC group. FBG, fasting blood glucose; OGTT, oral glucose tolerance test; AUC, area under the curve; INS, fasting blood insulin; HOMA-IR, homeostasis model assessment of insulin resistance; ISI, insulin sensitivity index; TC, total cholesterol; TG, triglycerides; HDL-C, high-density lipoprotein cholesterol.

Basic index analysis showed that compared with the C57BL/6J control group (NC group), KKAy mice showed remarkable metabolic abnormalities (*P*<0.01), including: accelerated body weight gain (**Figure 2A**), decreased serum 25(OH)D concentrations (**Figure 2B**), elevated FBG levels (**Figure 2C**), impaired glucose tolerance (**Figures 2D–F**), hyperinsulinemia evidenced by increased fasting insulin and HOMA-IR (**Figures 2G, H**), and dyslipidemia manifested by altered TC, LDL-C, and HDL-C levels (**Figures 2J–L**). Notably, 16-week VD_3_ supplementation significantly ameliorated all these metabolic abnormalities except for body weight and HDL-C changes (*P*<0.01).

### 3.2 VD_3_ supplementation preserves cardia structure and function in prediabetic mice

**Figure 3:**
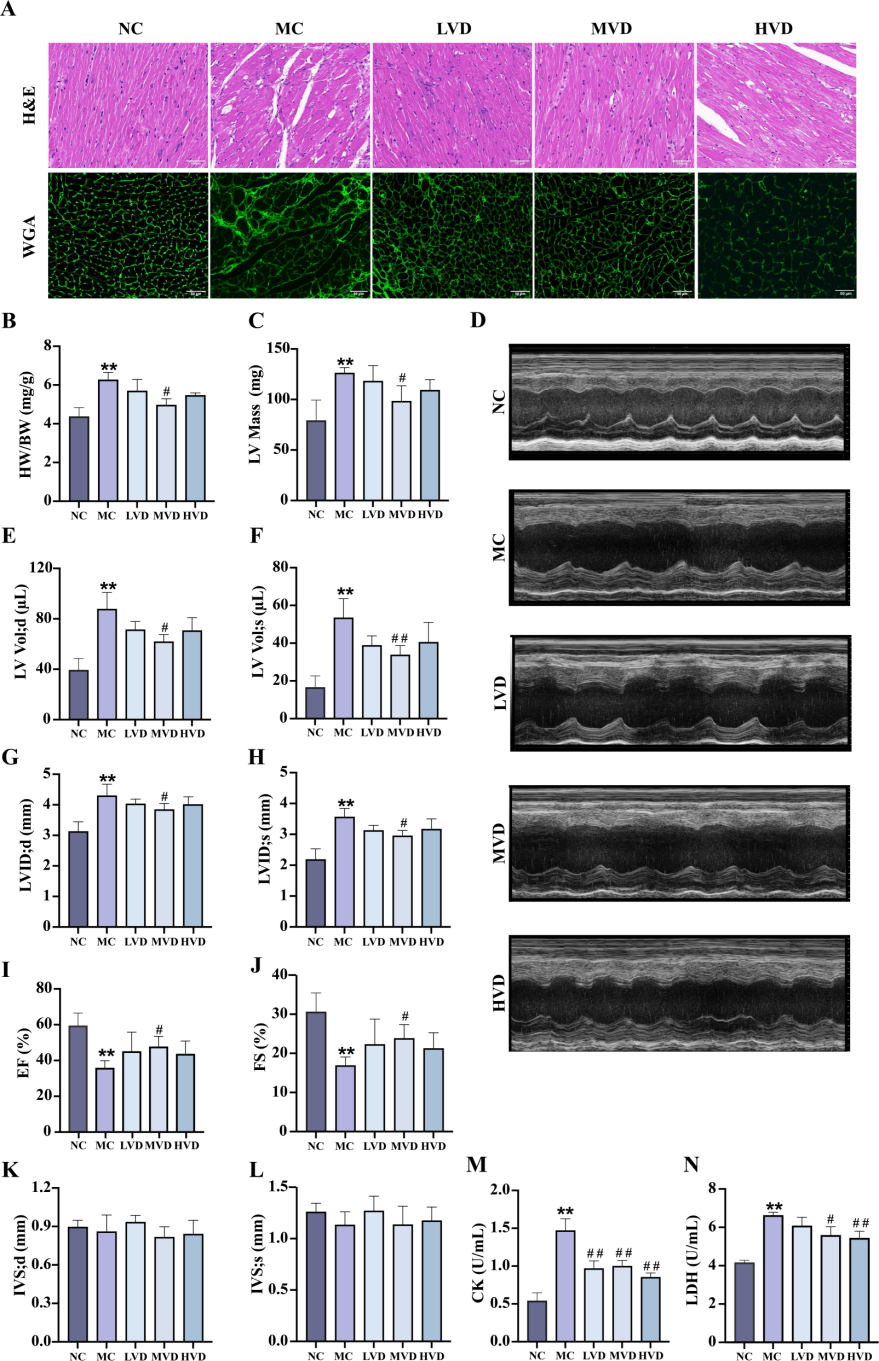
Effects of VD_3_ supplementation on myocardial structure and function in prediabetic mice. **(A)** H&E and WGA staining (n=3), scale bar=50 µm. **(B)** The heart indexes (n=3-6). **(C)** LV mass (n=5). **(D)** Echocardiography images (n=5). **(E-L)** Echocardiographic parameters: LV Vol;d, LV Vol;s, LVID;d, LVID;s, EF%, FS%, IVS;d and IVS;s (n=5). **(M)** Serum CK levels (n=6). **(N)** Serum LDH levels (n=6). Data are presented as Mean ± SD. ^*^ *P* < 0.05, ^**^ *P* < 0.01, compared with the NC group; ^#^ *P* < 0.05, ^##^ *P* < 0.01, compared with the MC group. CK, creatine kinase; LDH, lactate dehydrogenase.

Compared with the NC group, MC group mice exhibited significant cardiac remodeling (*P*<0.01), characterized by increased heart weight, cardiomyocyte hypertrophy, and marked disorganization of myocardial fibers with prominent vacuolization (**Figures 3A, B**). Echocardiographic assessment confirmed severe cardiac dysfunction in MC mice (*P*<0.01), demonstrating elevated LV mass (**Figure 3C**), increased end-diastolic and end-systolic LV volumes (LV Vol;d and LV Vol;s) (**Figures 3E, F**), and enlarged LV internal diameters during both diastole and systole (LVID;d and LVID;s) (**Figures 3G, H**). Concurrently, systolic function was significantly impaired, as evidenced by reduced ejection fraction (EF%) and fractional shortening (FS%) (**Figures 3I, J**) (*P*<0.01). VD_3_ intervention substantially ameliorated these pathological changes, yielding improved myocardial histoarchitecture with reduced hypertrophy, fewer structural discontinuities, and better organized fiber arrangement (*P*<0.01) (**Figures 3A, B**). Corresponding improvements were observed in echocardiographic parameters of ventricular structure and function. Additionally, VD_3_ treatment significantly attenuated the elevated serum levels of cardiac injury markers CK and LDH observed in MC mice (*P*<0.01) (**Figures 3M, N**). Collectively, these findings demonstrate that VD_3_ intervention effectively mitigates both structural and functional cardiac abnormalities in prediabetic mice.

### 3.3 VD_3_ supplementation attenuates cardiac ferroptosis in prediabetic mice

**Figure 4:**
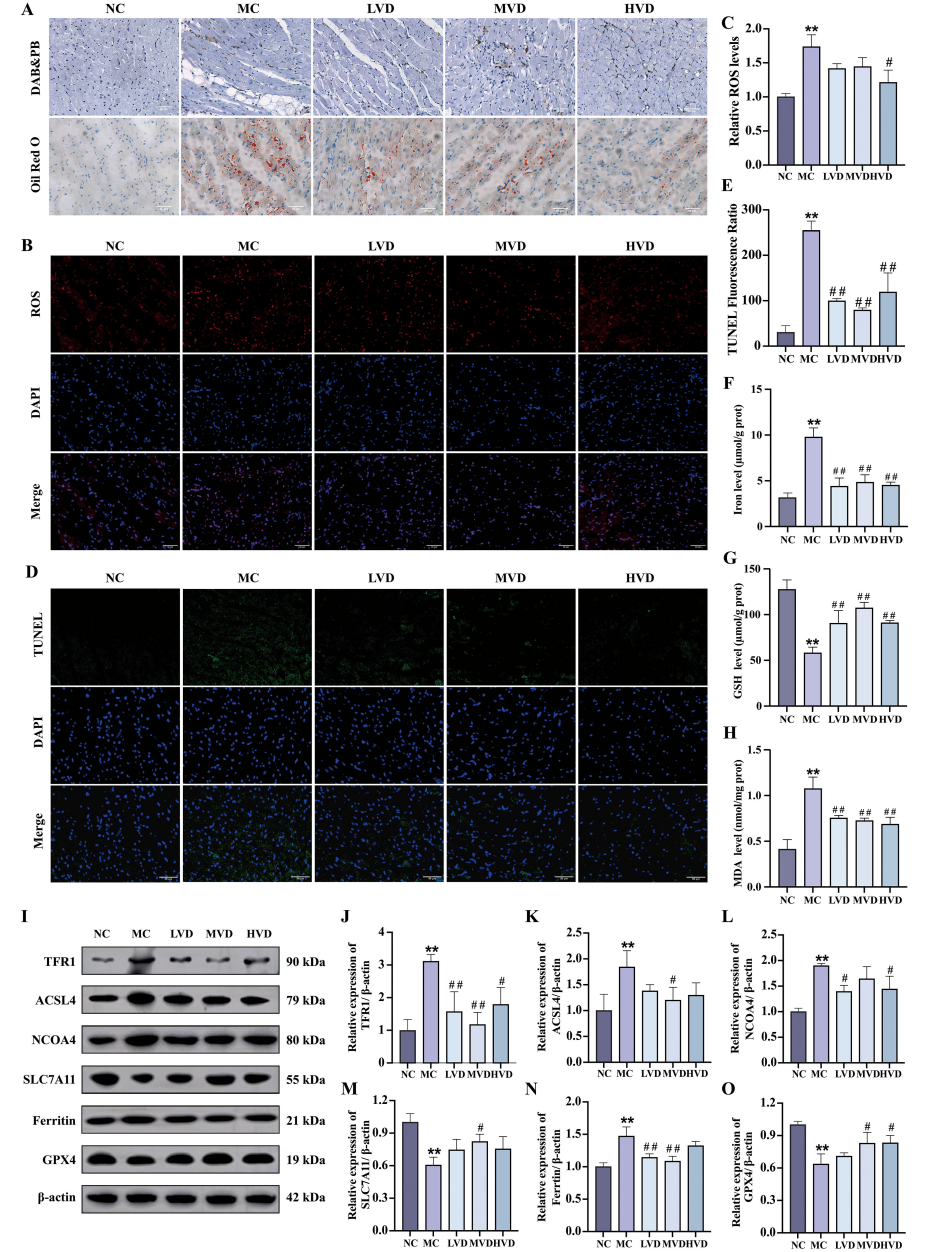
Effects of VD_3_ supplementation on ferroptosis in prediabetic mice. **(A)** Images of Prussian blue & DAB and Oil Red O staining of hearts tissue (n=3), scale bar=50 µm. **(B, C)** Representative fluorescence images and quantitative analysis of ROS measurement (n=3). **(D, E)** Quantitative analysis of TUNEL fluorescence ratio in myocardial tissues (n=3), scale bar=50 µm. **(F-H)** Analysis of Fe^2+^, GSH and MDA levels (n=5). **(I-O)** Representative western blot images and quantitative analysis of TFR1, ACSL4, NCOA4, SLC7A11, Ferritin and GPX4 protein expressions (n=3). Data are presented as Mean ± SD. ^*^ *P* < 0.05, ^**^ *P* < 0.01, compared with the NC group; ^#^ *P* < 0.05, ^##^ *P* < 0.01, compared with the MC group. ROS, reactive oxygen species; GSH, glutathione; MDA, malondialdehyde; TFR1, transferrin receptor protein 1; ACSL4, acyl-CoA synthetase long chain family member 4; NCOA4, nuclear receptor coactivator 4; SLC7A11, solute carrier family 7 member 11; GPX4, glutathione peroxidase 4.

Prediabetic mice exhibited characteristic hyperglycemia and hyperlipidemia, metabolic disturbances known to promote excessive ROS generation and lipid peroxidation, ultimately leading to ferroptosis. Histochemical analyses revealed striking pathological alterations in cardiac tissues of MC group mice, including abundant ferric hemoflavin-containing particles, excessive lipid accumulation, and marked ROS deposition (**Figures 4A–C**). These pathological features were significantly attenuated by VD_3_ intervention (*P*<0.05). The TUNEL assay confirmed increased cardiomyocyte death in MC mice, which was effectively mitigated by VD_3_ treatment (*P*<0.05). (**Figures 4D, E**). Quantitative assessments demonstrated that MC mice displayed elevated cardiac iron content and MDA levels, coupled with depleted GSH reserves compared to NC group (*P*<0.01) (**Figures 4F–H**). Western blot analysis further elucidated the molecular mechanisms underlying these observations: cardiac tissues from MC mice showed upregulated expression of ferroptosis-promoting proteins (TFR1, ACSL4, NCOA4, and Ferritin), while exhibiting downregulation of the ferroptosis inhibitors SLC7A11 and GPX4 (*P*<0.01) (**Figures 4I–O**). VD_3_ intervention consistently reversed these expression patterns (*P*<0.05). Collectively, these data demonstrate that ferroptosis contributes to cardiac pathology in prediabetic mice and that VD_3_ administration exerts protective effects against this form of programmed cell death.

### 3.4 VD_3_ supplementation suppresses ErbB4 and YAP activation in cardiac tissues of prediabetic mice

**Figure 5:**
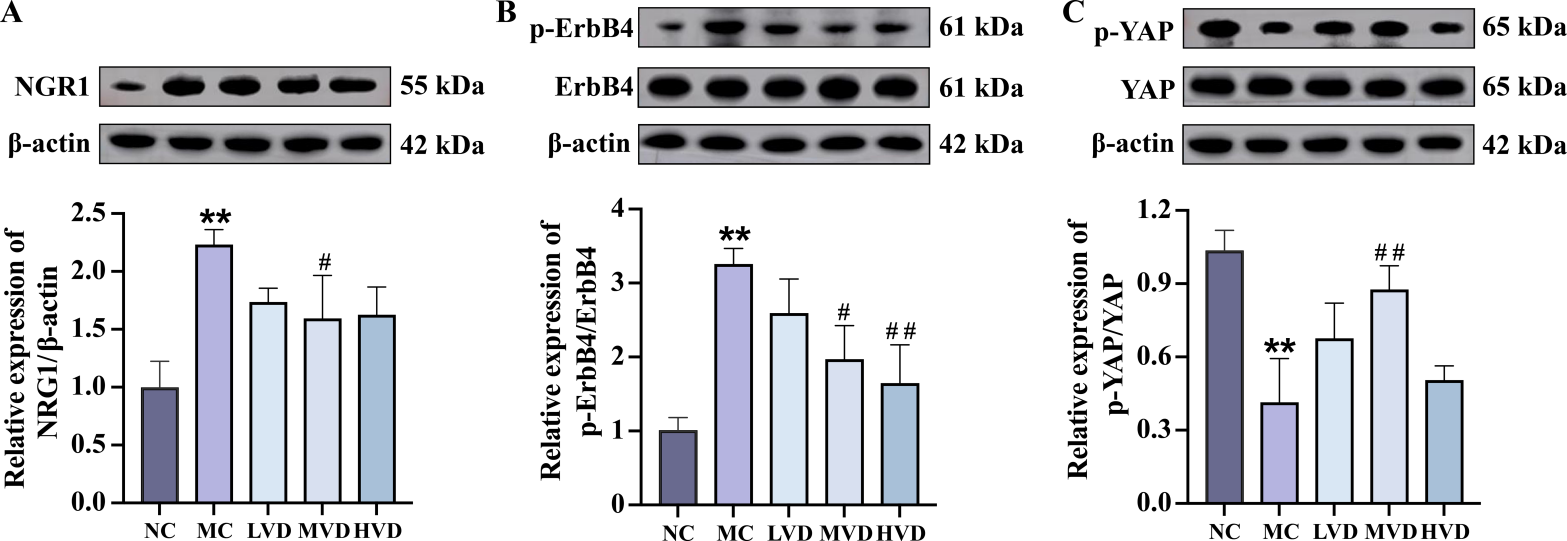
Effects of VD_3_ supplementation on ErbB4 and YAP activation in cardiac tissues of prediabetic mice. **(A-C)** Representative western blot images and quantitative analysis of NRG1, phosphorylated ErbB4, and phosphorylated YAP (n=3). Data are presented as Mean ± SD. ^*^ *P* < 0.05, ^**^ *P* < 0.01, compared with the NC group; ^#^ *P* < 0.05, ^##^ *P* < 0.01, compared with the MC group. NRG1, neuregulin 1; ErbB4, receptor tyrosine-protein kinase erbB-4; YAP, yes-associated protein.

To further delineate the molecular mechanisms responsible for the cardioprotective effects of VD_3_, the expression of key signaling proteins in myocardial tissues were evaluated. Quantitative analysis revealed a significant upregulation of both NRG1 and phosphorylated ErbB4 (p-ErbB4) in MC mice compared to NC group (*P*<0.01) (**Figures 5A, B**), an effect that was substantially attenuated following VD_3_ intervention (*P*<0.05). Notably, VD_3_ treatment also induced a marked increase in phosphorylated YAP (p-YAP) levels, indicating effective activation of YAP (*P*<0.05) (**Figure 5C**). Collectively, these findings demonstrate that VD_3_ ameliorates prediabetic myocardial injury by downregulating NRG1 expression and subsequently affecting the activation of both ErbB4 and YAP signaling cascades.

### 3.5 Inhibition of ferroptosis alleviates HGPA -induced cardiomyocyte injury

To further elucidate the mechanisms underlying cardiomyocyte injury, an *in vitro* model was established using H9c2 cardiomyocytes exposed to HGPA. As depicted in supplementary **Figure 1**, exposure to a combination of 33.3 mM HG and 0.25 mM PA for 24h (designated as HGPA) significantly reduced cell viability (*P*<0.01). Notably, pre-treatment with 10 nM 1,25(OH)_2_D_3_ for 2h remarkably enhanced cell viability followed by co-culture with HGPA for 24h (*P*<0.01).

**Figure 6:**
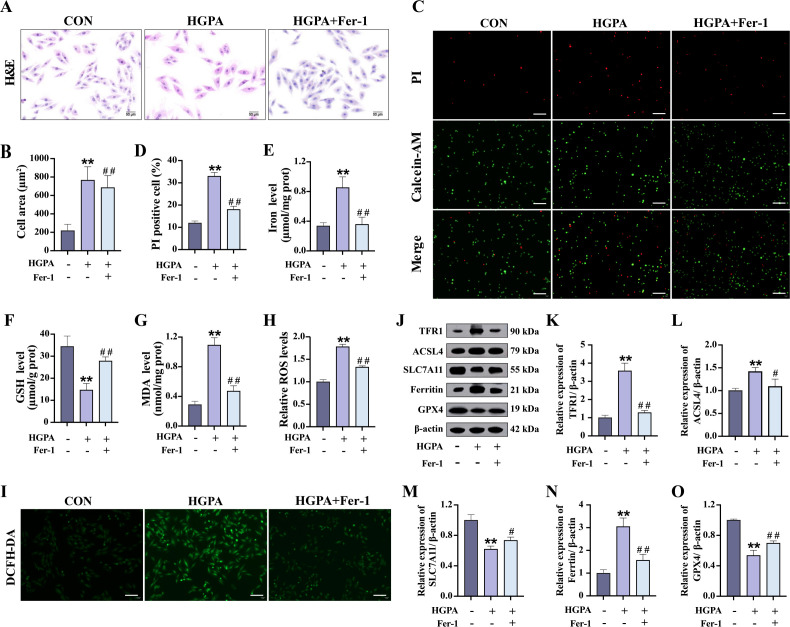
Effect of ferroptosis inhibition on HGPA-induced cardiomyocyte injury. **(A-D)** Myocardial hypertrophy and cell death status were assessed using images of H&E and PI/Calcein-AM staining (n=3), scale bar=50 µm. **(E-G)** Fe^2+^, GSH and MDA levels in different groups (n=6). **(H, I)** Representative fluorescence images and quantitative analysis of DCFH-DA staining for ROS measurement in H9c2 cells (n=3), scale bar=50 µm. **(J-O)** Representative western blot images and quantitative analysis of TFR1, ACSL4, SLC7A11, Ferritin and GPX4 (n=3). Data are presented as Mean ± SD. ^*^ *P* < 0.05, ^**^ *P* < 0.01, compared with the CON group; ^#^ *P* < 0.05, ^##^ *P* < 0.01, compared with the HGPA group. GSH, glutathione; MDA, malondialdehyde; ROS, reactive oxygen species; TFR1, transferrin receptor protein 1; ACSL4, acyl-CoA synthetase long chain family member 4; SLC7A11, solute carrier family 7 member 11; GPX4, glutathione peroxidase 4.

Subsequently, we explored whether inhibiting ferroptosis could alleviate cardiomyocyte injury induced by HGPA. *In vitro*, cells were initially exposed to 1 μM Fer-1 for 2h, followed by co-incubation with HGPA for 24 h. A significant increase in the cross-sectional area was observed in HGPA+Fer-1 cells compared to HGPA group (**Figures 6A, B**). Additionally, the number of dead cells was notably decreased (**Figures 6C, D**), indicating a degree of recovery from damage. Furthermore, elevated levels of Fe^2+^, MDA, and ROS, along with a substantial decrease in GSH levels, were detected in HGPA cells compared to normal cells (**Figures 6E–I**). Notably, these alterations were reversed upon application of the Fer-1. Western blot analysis was subsequently performed to assess key proteins implicated in ferroptosis. The results revealed a substantial upregulation in the relative expression levels of TFR1, ACSL4, and Ferritin, along with a notable reduction in the relative expression of SLC7A11 and GPX4 following HGPA exposure (**Figures 6J–O**). Intriguingly, these changes were reversed upon treatment with the Fer-1. This study thus delineates the induction of ferroptosis in H9c2 cells by HGPA and highlights the potential of ferroptosis inhibition in mitigating HGPA-triggered cardiomyocyte injury.

### 3.6 Phospho-ErbB4 inhibitor modulates YAP-mediated ferroptosis and hypertrophy in H9c2 cardiomyocytes

**Figure 7:**
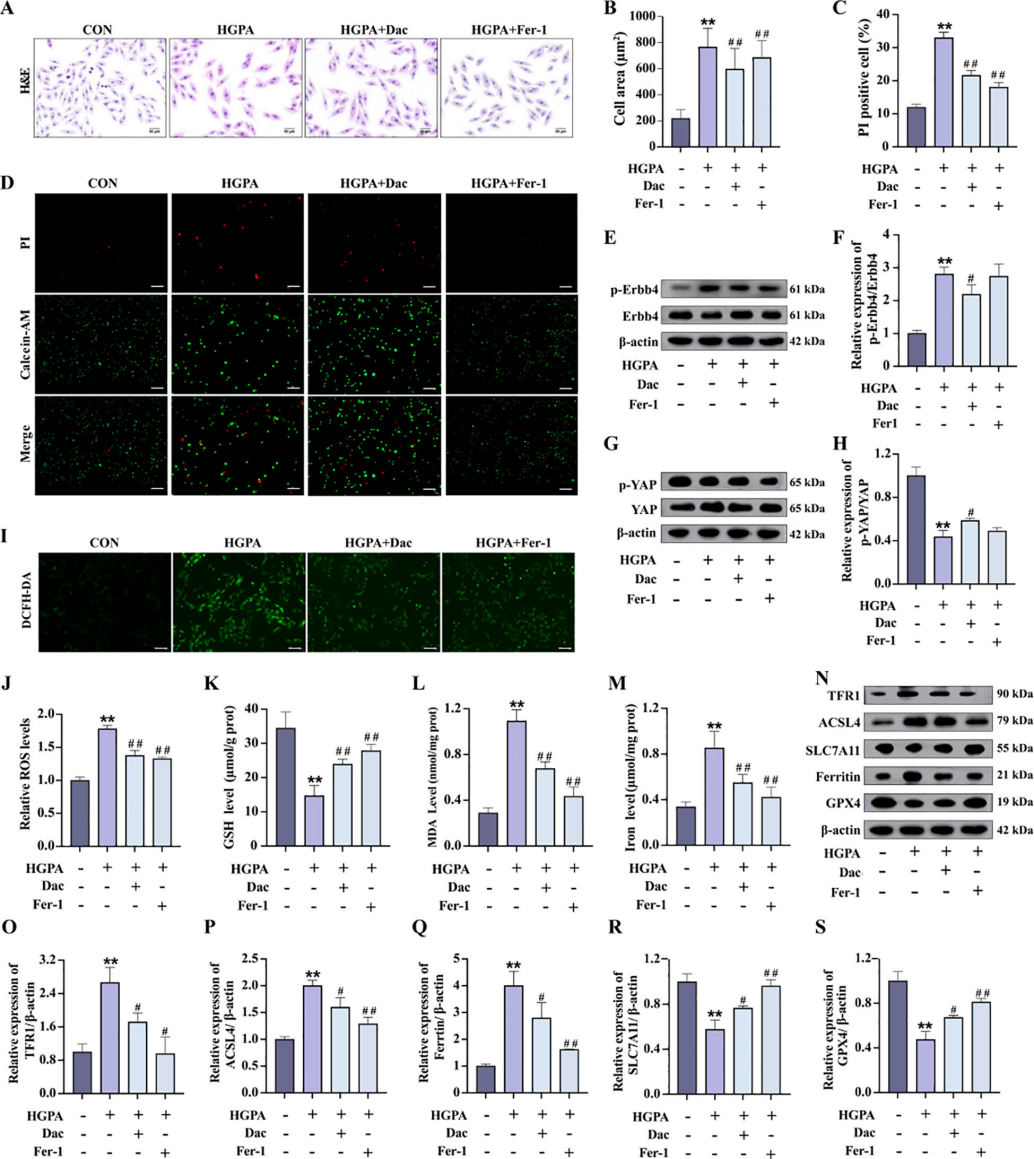
p-ErbB4 inhibitor regulate YAP-mediated ferroptosis in H9c2 cardiomyocytes. **(A-D)** Myocardial hypertrophy and cell death status were assessed by Images of H&E and PI staining (n=3), scale bar=50 µm. **(E-H)** Representative western blot images and quantitative analysis of NRG1, phosphorylated ErbB4, and phosphorylated YAP (n=3). **(I-J)** Representative fluorescence images and quantitative analysis of DCFH-DA staining for ROS measurement in H9c2 cells (n=3), scale bar=50 µm. **(K-M)** Analysis of Fe^2+^, GSH and MDA levels in H9c2 cells (n=6). **(N-S)** Representative western blot images and quantitative analysis of TFR1, ACSL4, SLC7A11, Ferritin and GPX4 (n=3). Data are presented as Mean ± SD. ^*^ *P* < 0.05, ^**^ *P* < 0.01, compared with the CON group; ^#^ *P* < 0.05, ^##^ *P* < 0.01, compared with the HGPA group. ErbB4, receptor tyrosine-protein kinase erbB-4; YAP, yes-associated protein; GSH, glutathione; MDA, malondialdehyde; ROS, reactive oxygen species; TFR1, transferrin receptor protein 1; ACSL4, acyl-CoA synthetase long chain family member 4; SLC7A11, solute carrier family 7 member 11; GPX4, glutathione peroxidase 4.

During the progression of prediabetic myocardial injury, our investigations revealed concurrent ErbB4 activation, YAP activity suppression, and ferroptosis occurrence. To elucidate the precise interrelationship among these three factors, we conducted *in vitro* experiments involving pretreatment with 73.7 nM Dac for 2h followed by 24-h co-culture with HGPA to specifically inhibit ErbB4 phosphorylation and monitor subsequent changes. Comparative analysis demonstrated that Dac-treated cells exhibited significantly reduced cell mortality and markedly diminished cellular cross-sectional area (**Figures 7A–D**) relative to HGPA-treated cells. Western blot analysis confirmed effective ErbB4 phosphorylation inhibition (**Figures 7E, F**), which was accompanied by a substantial elevation in phosphorylated YAP levels (**Figures 7G, H**) and concurrent attenuation of ferroptosis markers (**Figures 7I–S**). Notably, the phosphorylation profiles of both ErbB4 and YAP showed no statistically significant differences between Fer-1-treated and Dac-treated groups. These collective findings establish a hierarchical relationship wherein ErbB4 operates upstream of YAP, and its reduced phosphorylation state appears to mitigate YAP-mediated ferroptotic processes in the context of prediabetic myocardial injury.

### 3.7 1,25(OH)_2_D_3_ inhibits ErbB4/YAP-dependent ferroptosis and attenuates myocardial injury

**Figure 8:**
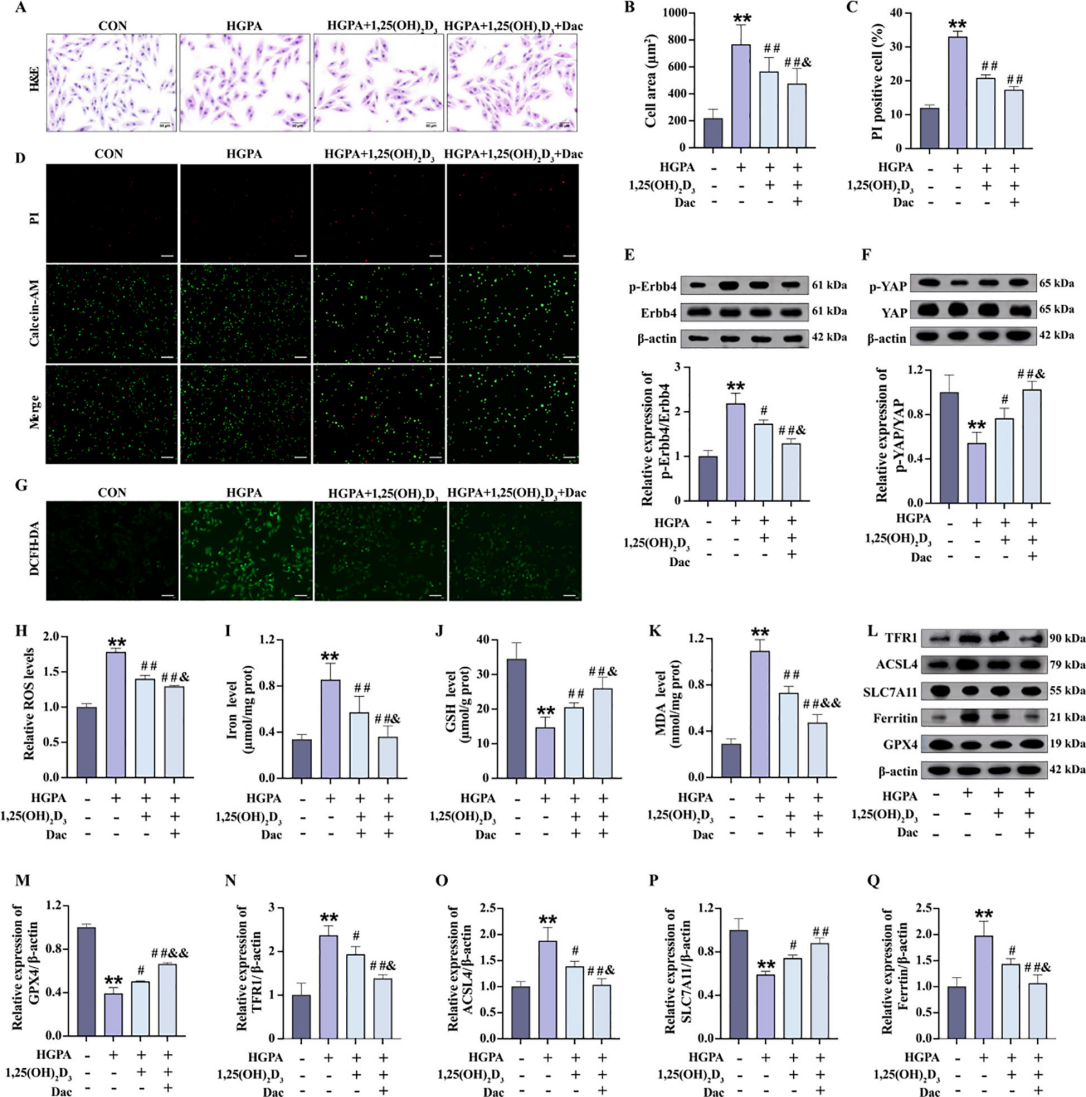
1,25(OH)_2_D_3_ inhibits ErbB4/YAP-mediated ferroptosis and rescues myocardial injury. **(A-D)** Myocardial hypertrophy and cell death status were assessed by images of H&E and PI staining (n=3), scale bar=50 µm. **(E, F)** Representative western blot images and quantitative analysis of NRG1, phosphorylated ErbB4, and phosphorylated YAP (n=3). **(G, H)** Representative fluorescence images and quantitative analysis of DCFH-DA staining for ROS measurement in H9c2 cells (n=3), scale bar=50 µm. **(I-K)** Analysis of Fe^2+^, GSH and MDA levels in H9c2 cells (n=6). **(L-Q)** Representative western blot images and quantitative analysis of TFR1, ACSL4, SLC7A11, Ferritin and GPX4 (n=3). Data are presented as Mean ± SD. ^*^ *P* < 0.05, ^**^ *P* < 0.01, compared with the CON group; ^#^ *P* < 0.05, ^##^ *P* < 0.01, compared with the HGPA group; ^&^ *P* < 0.05, ^&&^ *P* < 0.01, compared with the HGPA+1,25(OH)_2_D_3_ group. ErbB4, receptor tyrosine-protein kinase erbB-4; YAP, yes-associated protein; GSH, glutathione; MDA, malondialdehyde; ROS, reactive oxygen species; TFR1, transferrin receptor protein 1; ACSL4, acyl-CoA synthetase long chain family member 4; SLC7A11, solute carrier family 7 member 11; GPX4, glutathione peroxidase 4.

Our previous *in vivo* study demonstrated a decrease in p-ErbB4 expression following VD_3_ intervention. Subsequently, we explored whether VD mitigates myocardial injury by modulating ErbB4/YAP-mediated ferroptosis. Therefore, *in vitro*, we added 10 nM 1,25(OH)_2_D_3_ and 73.7 nM Dacomitinib for 2h pretreatment followed by 24h co-culture with HGPA [1,25(OH)_2_D_3_+Dac]. Compared with HGPA group, HGPA+1,25(OH)_2_D_3_ group showed a significant decrease in phosphorylated ErbB4 levels (**Figure 8E**), a significant increase in phosphorylated YAP levels (**Figure 8F**), and an inhibition of ferroptosis (**Figures 8G-Q**). Compared with HGPA+1,25(OH)_2_D_3_ group, the above indicators of HGPA+1,25(OH)_2_D_3_+Dac group were reversed more significantly, the cell cross-sectional area was significantly reduced (**Figures 8A, B**), and the number of dead cells was reduced (**Figures 8C, D**). The above analyses suggest that 1,25(OH)_2_D_3_ inhibits ErbB4/YAP-mediated ferroptosis and rescues myocardial injury, and that p-ErbB4 inhibitor enhance the therapeutic effect of 1,25(OH)_2_D_3_ on myocardial injury.

## 4 Discussion

Accumulating evidence indicates that cardiac injury initiates during the prediabetic stage (4, 5). Without timely intervention, this early injury may progress to diabetic cardiomyopathy - a distinct form of heart failure with potentially fatal consequences (1). The pathogenesis of diabetic cardiomyopathy is complex and multifactorial, and currently, no effective preventive or therapeutic strategies are available. Notably, observational studies have demonstrated an inverse correlation between serum 25(OH)D levels and cardiovascular risk in prediabetic patients (20). Additionally, VD status has been associated with cardiac autonomic function and metabolic regulation in prediabetes (21). These findings motivated our investigation into the cardioprotective effects of VD in prediabetic states, utilizing both *in vivo* and *in vitro* models.

The therapeutic potential of VD in prediabetes remains controversial. While some studies report that VD supplementation improves insulin sensitivity and reduces diabetes risk in VD-deficient prediabetic rodents, others have found no significant improvement in β-cell function indices following 24 months of VD_3_ treatment in prediabetic patients (22–24). Our experimental data reveal that HFD-fed KKAy mice developed characteristic prediabetic features including hypovitaminosis D, hyperglycemia, dyslipidemia, glucose intolerance, insulin resistance, and obesity. Notably, a 16-week VD_3_ intervention effectively ameliorated these metabolic disturbances, suggesting that VD_3_ supplementation enhance insulin sensitivity and potentially delay diabetes onset in prediabetic individuals.

The cardiac pathology of diabetes spans structural abnormalities, functional impairments, and metabolic dysregulation (25, 26). In our study, echocardiographic analysis revealed that HFD-fed KKAy mice developed significant left ventricular dilation (increased volume and internal diameter), hypertrophy (increased mass), and systolic dysfunction (reduced EF and FS). These echocardiographic findings were corroborated by elevated serum cardiac enzymes (CK, LDH) and histopathological evidence of cardiomyocyte hypertrophy. VD_3_ treatment significantly attenuated these pathological changes. These results are in line with clinical studies reporting that high-dose VD_3_ supplementation can reduce left atrial enlargement and improve cardiovascular risk profiles in prediabetic patients (27, 28).

Our mechanistic investigation further revealed that KKAy mice exhibited increased myocardial ROS production and lipid peroxidation—hallmarks of ferroptosis (29, 30). Ferroptosis, an iron-dependent form of regulated cell death, has recently emerged as a promising therapeutic target in cardiovascular diseases, including diabetic cardiomyopathy (31, 32). Among oxidative stress biomarkers, GSH and MDA are widely recognized indicators of ferroptotic activity (33). GSH, the most abundant intracellular antioxidant, neutralizes lipid peroxides via the GPX4 pathway (34). Its depletion weakens cellular antioxidant defenses and is a defining feature of ferroptosis. Conversely, MDA, a reactive byproduct of lipid peroxidation, reflects oxidative damage to cellular membranes (35). In our model, prediabetic mice showed reduced myocardial GSH levels and elevated MDA levels, indicating ferroptosis and oxidative stress. Importantly, VD_3_ treatment significantly restored GSH levels and decreased MDA accumulation, suggesting a protective effect of VD against oxidative stress–induced cardiomyocyte injury in prediabetic states. To functionally validate these findings, we used Ferrostatin-1, a specific ferroptosis inhibitor, in HGPA-treated H9c2 cardiomyocytes. Ferrostatin-1 effectively attenuated glycolipotoxicity-induced cardiomyocyte hypertrophy and cell death—findings consistent with previous reports by Wang et al. (32, 36). Moreover, while previous studies have shown that VD can regulate ferroptosis in various organs, including the heart, by inhibiting iron accumulation and lipid peroxidation (37–40), our data provide new evidence supporting a role for VD in modulating ferroptosis as a mechanism of cardioprotection in prediabetic models.

ErbB4, a key member of the EGFR family, is predominantly expressed in cardiac tissue and plays essential roles in cardiac development, physiological maintenance, as well as functions in the mammary gland and nervous system (41–43). Recent evidence has implicated ErbB4 in cardiac pathophysiology. For example, cardiac endothelial-specific ErbB4 deficiency has been shown to attenuate myocardial hypertrophy and fibrosis under stress (16), while ErbB4 activation can promote cardiomyocyte hypertrophy (15). In our study, cardiac tissue from HFD-induced KKAy mice displayed significant ErbB4 activation along with increased expression of its ligand, neuregulin-1 (NRG1). We found that this NRG1/ErbB4 signaling axis contributes to the development of myocardial hypertrophy during prediabetic cardiac injury. Furthermore, we observed activation of Yes-associated protein (YAP), a nuclear effector of the Hippo pathway, in these mice, which exhibited marked left ventricular dilation—a hallmark of hypertrophic remodeling. These observations are consistent with prior studies showing YAP’s involvement in both compensatory hypertrophy following acute pressure overload (44) and pathological cardiac remodeling (45, 46). Importantly, VD_3_ treatment effectively suppressed activation of both ErbB4 and YAP. This is in line with reports that VD analogs can bind ErbB4 (47) and modulate TGF-β-induced YAP phosphorylation (48, 49). These findings suggest that VD’s cardioprotective effects in prediabetes may, at least in part, be mediated via inhibition of the ErbB4/YAP signaling axis.

In our model, the MC group exhibited three key molecular changes: enhanced ErbB4 activation, suppressed YAP activity, and increased ferroptosis in cardiac tissues. Based on these observations, we hypothesized that ErbB4 signaling might serve as a regulatory node linking cardiac stress to ferroptosis during prediabetic injury. To test this, we conducted *in vitro* experiments using phospho-ErbB4 (p-ErbB4) and ferroptosis inhibitors in HGPA-treated H9c2 cells. Our results support a model in which ErbB4 acts upstream of YAP and potentially modulates ferroptosis by disrupting YAP-mediated signaling. Notably, treatment with 1,25(OH)_2_D_3_ mimicked the effects of p-ErbB4 inhibition, suppressing the ErbB4/YAP axis and reducing ferroptosis-related cellular injury. These *in vitro* findings further validate our *in vivo* results and suggest that VD may mitigate prediabetic cardiac injury through modulation of the ErbB4/ferroptosis axis. The therapeutic relevance of this pathway is highlighted by the enhanced cardioprotective effects observed when combining 1,25(OH)_2_D_3_ with a p-ErbB4 inhibitor.

In summary, our study demonstrates that VD exerts beneficial effects on prediabetic cardiac injury. VD_3_, the inactive precursor form, undergoes sequential hydroxylation—first in the liver to form 25-hydroxyvitamin D_3_ [25(OH)D_3_], and then in the kidney to produce 1,25(OH)_2_D_3_, the biologically active form. We used VD_3_ supplementation *in vivo* to assess systemic effects, and 1,25(OH)_2_D_3_ *in vitro* to directly probe cellular mechanisms. This dual approach enabled us to more precisely delineate the mechanistic basis of VD-mediated cardioprotection under prediabetic conditions.

Despite these promising findings, several limitations must be acknowledged. The *in vivo* environment is inherently complex, involving multiple interacting factors that may influence outcomes. Therefore, additional validation using population-based studies or ErbB4 knockdown/knockout animal models is necessary to confirm the proposed mechanisms. Moreover, our current investigation primarily focuses on the anti-hypertrophic effects of VD. Future studies should further explore its potential pharmacological impact on other pathological aspects of diabetic cardiomyopathy, including inflammation, myocardial fibrosis, and diastolic dysfunction.

## 5 Conclusions

Our study reveals for the first time the relationship between ErbB4 and ferroptosis in prediabetic cardiopathy, which provides novel evidence that VD can ameliorate diabetic cardiac injury, and that the combination of VD and p-ErbB4 inhibitor may be a feasible clinical strategy for treating prediabetic myocardial injuries.

## Data Availability

The raw data supporting the conclusions of this article will be made available by the authors, without undue reservation.
