# Supplementary material for: Vitamin D ameliorates prediabetic cardiac injure via modulation of the ErbB4/ferroptosis signaling axis
Source: Front Immunol. 2025 Jul 17;16:1626295. doi: 10.3389/fimmu.2025.1626295 (PMC12310489; doi:10.3389/fimmu.2025.1626295)
Supplement: Supplementary file 1 [file SupplementaryFile1.docx]

**Supplementary Figure**

**
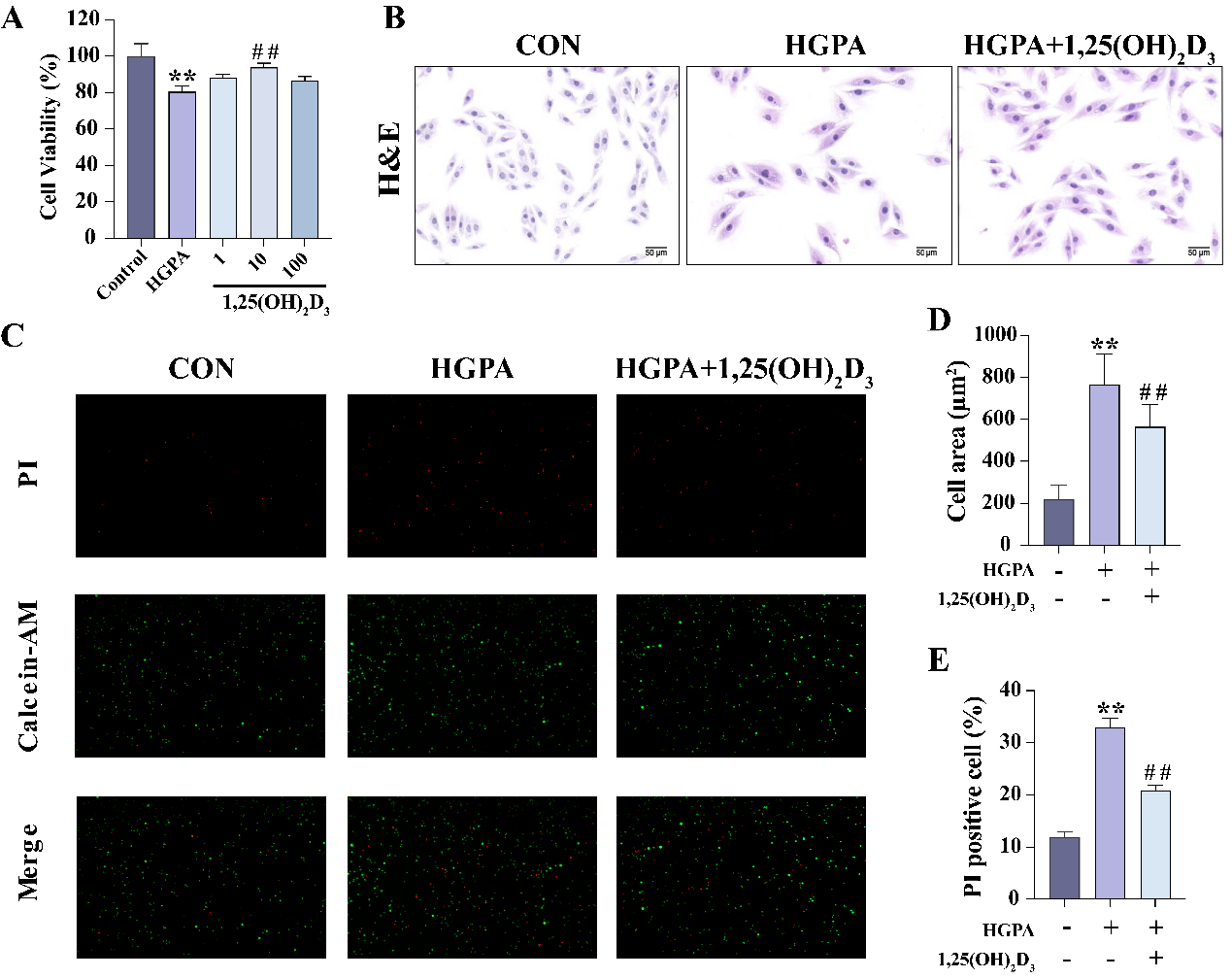
**

**Fig. 1.** 1,25(OH)_2_D_3_ provides protection against cell damage induced by HGPA. (A) Cell viability analysis following various interventions. (B-E) Myocardial hypertrophy and cell death status were evaluated by images of H&E and PI/Calcein-AM staining. Data were presented as Mean ± SD. *P < 0.05, **P < 0.01, compared with the CON group; #P < 0.05, ##P < 0.01, compared with the HGPA group.
